# Pregnancy related protection against breast cancer depends on length of gestation

**DOI:** 10.1038/sj.bjc.6600453

**Published:** 2002-08-01

**Authors:** L J Vatten, P R Romundstad, D Trichopoulos, R Skjærven

**Affiliations:** Department of Community Medicine and General Practice, The Norwegian University of Science and Technology, Trondheim, Norway; Department of Epidemiology, Harvard School of Public Health, Boston, Massachusetts, USA; The Medical Birth Registry and Section for Medical Statistics, University of Bergen, Norway

**Keywords:** pregnancy, length of gestation, breast cancer

## Abstract

In a prospective study of 694 657 parous women in Norway, 5474 developed breast cancer after their first birth. If the first pregnancy lasted less than 32 weeks, the risk was 22% (95% confidence interval, −3% to 53%) greater than after a pregnancy of 40 weeks or more, with a significant declining trend in risk (*P* for trend=0.02).

*British Journal of Cancer* (2002) **87**, 289–290. doi:10.1038/sj.bjc.6600453 www.bjcancer.com

© 2002 Cancer Research UK

## 

Pregnancies generally convey protection against breast cancer, and the degree of protection depends on the woman's age at childbearing (Trichopoulos *et al*, 1983). An early age at first birth provides a particularly strong protective effect that is independent of the effects of subsequent pregnancies (Rosner *et al*, 1994). This effect has been attributed to terminal differentiation of mammary cells brought about by the hormonal milieu of pregnancy (Russo and Russo, 1999). It has been shown that pregnancies that are spontaneously or intentionally interrupted in early gestation do not provide protection against breast cancer (Melbye *et al*, 1997). There have been, however, no data directly addressing the hypothesis that the protective effect of a pregnancy depends on the length of gestation across the whole range of gestational age.

In this study we have combined information from two nationwide health registries in Norway in order to examine whether length of gestation is related to breast cancer risk. Data were derived from the Medical Birth Registry that comprises all births since 1967, and the Norwegian Cancer Registry, which has registered incident cancers since 1953. Midwives and doctors have to fill in a standardised form to notify the Birth Registry about each birth that takes place in the country, and the reporting of new cancers to the Cancer Registry is also mandatory. Length of gestation, based on the last menstrual period, was recorded in more than 90% of all pregnancies, and included pregnancies lasting from 154 to more than 300 days of gestation. In the analysis, length of gestation was categorised into four separate categories.

We used the unique national identification number to link women registered at the Medical Birth Registry and the national Cancer Registry to identify women who had developed breast cancer subsequent to giving birth. A total of 695 873 women had been registered with a first pregnancy between 1967 and the end of 1998, for whom length of gestation and infant birth weight had been recorded. Of those, 1216 were excluded from analysis, either because they had a diagnosis of cancer recorded prior to their first birth, or because they had emigrated and could not be traced. Thus, we have followed 694 657 women from their first birth in 1967 or later until the diagnosis of cancer, death from any cause, or to the end of follow-up (December 31, 1998), whichever occurred first. We examined whether breast cancer risk differed between women who had different length of gestation in their first pregnancy. In the analysis, we adjusted for attained age (nine categories), calendar period of diagnosis (three categories), age at first birth (five categories), and number of subsequent births (five categories), using Poisson regression modelling (EPICURE, Seattle, WA, USA; Hirosoft Int Corp, 1993).

We found, as expected (Trichopoulos *et al*, 1983; Rosner *et al*, 1994), a gradual increase in breast cancer risk with increasing age at first birth (Table 1

**Table 1:**
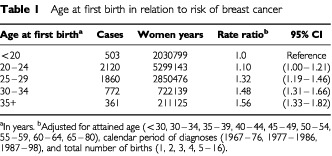
Age at first birth in relation to risk of breast cancer

), and an additional protection with increasing number of births (data not shown). However, increased length of gestation in the first pregnancy was strongly and independently related to reduction in breast cancer risk (Table 2

**Table 2:**
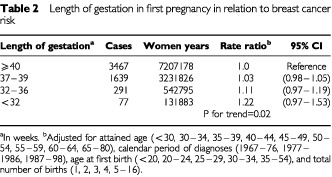
Length of gestation in first pregnancy in relation to breast cancer risk

). A relatively short pregnancy of less than 32 weeks was associated with a 22% (95% confidence interval, −3 to 53%) higher risk of breast cancer than a full term pregnancy of 40 weeks or more. The increase in risk related to shorter length of gestation displayed, a consistent pattern across the range of gestational age (*P* for trend=0.02).

This large prospective study is based on linkage between reliable data from two established national registries (Irgens, 2000), and it is unlikely that selection or information bias could have influenced the results. Our findings indicate that the protection against breast cancer depends on the duration of exposure to pregnancy, and that there is no threshold effect after a length of gestation of about 30 weeks. Combined with the evidence that pregnancies stimulate the terminal differentiation of mammary cells (Russo and Russo, 1999), these findings could be useful for our understanding of how breast cancer occurs and how it could be prevented.
